# Humanoid Robot–Assisted Support for Health Care in Older Adults: Systematic Scoping Review

**DOI:** 10.2196/83849

**Published:** 2026-03-11

**Authors:** Lei Cui, Yufei Li, Xinyao Yang, Xue Liu, Like Zhang, Lili Hou

**Affiliations:** 1 School of Nursing, Shanghai Jiao Tong University, Shanghai, China; 2 Pancreatic Center, The First Affiliated Hospital with Nanjing Medical University, Nanjing, China; 3 Department of Nursing, Shanghai Ninth People's Hospital, Shanghai Jiao Tong University School of Medicine, No. 639, Zhizaoju Road, Huangpu District, Shanghai, 200011, China, 86 13816033620

**Keywords:** humanoid robots, older, older adults, robotics, scoping review, socially assistive robots

## Abstract

**Background:**

Humanoid robots offer a promising solution to the growing burden of care for older adults. However, existing evidence on their applications for general aging populations remains fragmented and lacks systematic synthesis.

**Objective:**

This scoping review aimed to examine the literature on humanoid robot–assisted support for health care in older adults and identify gaps in the literature to guide future research.

**Methods:**

The methodological framework by Arksey and O’Malley was used to conduct this scoping review. We conducted a comprehensive search in 8 databases, including IEEE Xplore Digital Library, CINAHL, Cochrane Library, EMBASE, PubMed, Scopus, Web of Science, and OpenGrey Repository, covering literature published up to April 30, 2025. The reference lists of key texts were examined, and citation chaining was conducted. Two independent reviewers examined all full articles for fitness with the eligibility criteria and extracted data elements. The study findings were then summarized, coded, and analyzed using the PAGER (Patterns, Advances, Gaps, Evidence for practice, and Research recommendations) framework.

**Results:**

A total of 32,477 articles were retrieved, 59 of which were included in this review. The majority (49/59, 83%) were conducted in real-world settings. Methodologically, 34 studies (34/59, 58%) had small sample sizes (n≤25), with study designs comprising 26 quantitative (26/59, 44%), 22 mixed method (22/59, 37%), and 11 qualitative (11/59, 19%) approaches. Participant characteristics revealed female predominance (>50%) in 32 studies (32/59, 54%), while 27 studies (27/59, 46%) included participants with cognitive impairment. Through PAGER framework analysis, we identified 4 key patterns: (1) effects, perceptions, and experiences of humanoid robots; (2) preferences, expectations, and facilitators for humanoid robots; (3) implementation barriers and challenges; and (4) determinants of user experiences and outcomes.

**Conclusions:**

This scoping review demonstrates the promising yet methodologically constrained potential of humanoid robots in health care for older adults while highlighting key challenges in their practical implementation. Successful integration will require addressing technical limitations, user acceptance barriers, and systemic adoption challenges.

## Introduction

Population aging has intensified worldwide, constituting a critical societal challenge. According to the United Nations World Population Prospects 2024 report, the proportion of individuals aged ≥65 years doubled from 5% in 1960 to 10% in 2024, and projections suggest this figure will surpass one-third of the population in the European Union, China, and Japan by 2050 [1]. A study tracking staffing trends since the onset of the COVID-19 pandemic found that 99% of nursing homes and 96% of assisted living facilities in the United States continued to face significant staffing shortages [2]. This study further indicated that 58% of nursing homes were limiting admissions due to lack of staff, with 78% expressing concern that workforce shortages might force them to close [2]. Despite measurable recovery observed in a 2024 follow-up study, employment levels in skilled nursing facilities remained 0.1% below projected benchmarks [3]. Concurrently, informal care systems face growing pressures, with Europe’s care burden projected to increase 49.7% by 2050 [4], while caregivers endure physical, emotional, and financial strain [5]. The macroeconomic impact is equally concerning, with each 1% increase in care dependency reducing gross domestic product growth by 0.083% through workforce attrition [6]. These intersecting crises in both professional and family-based care systems highlight the critical need for innovative support solutions in older adult care.

Humanoid robots present a promising avenue to address these systemic challenges. Since its inception in the 1970s, humanoid robotics has undergone a remarkable evolution, transitioning from rudimentary mechanical mimics to sophisticated platforms capable of complex social interactions [7]. Their anthropomorphic architecture—featuring human-like torsos, limbs, and movement patterns—confers unique advantages in care contexts for older adults. This biomimetic design enhances environmental navigation in human-centric spaces, fosters natural communication through familiar gestural cues, and improves social acceptance compared with nonanthropomorphic alternatives [7]. These attributes make humanoid robots a compelling platform for exploring the integration of physical and psychosocial support in older adult care. Empirical studies have begun to investigate these applications across multiple care domains. For example, the Pepper platform has been evaluated in the domains of cognitive training [8], object transfer [9], and social communication [10]. Similarly, the Nao robot has been studied in contexts such as health education delivery [11] and social interaction therapies [12] for older adults.

The field of robotics and gerontechnology has seen significant growth in research focused on developing care robots to support older adults and their caregivers [13]. Although existing reviews have examined robotic applications in health care, they present several important limitations that warrant addressing to advance the field. First, by conflating anthropomorphic and nonanthropomorphic robotic systems without distinguishing their distinct capabilities [14-16], prior syntheses obscure the unique potential of humanoid forms for fostering natural social engagement—a critical dimension in older adult care. Second, many reviews incorporate heterogeneous populations spanning multiple age groups and health conditions [15], thereby diluting findings that are specifically relevant to the physiological, cognitive, and psychosocial needs of the older adult population. Third, existing analyses often focus narrowly on isolated aspects, such as single-function applications, implementation challenges, or ethical issues in isolation [14,16-18], while neglecting comprehensive, multidimensional assessments of how multifunctional robotic systems operate within integrated older adult care environments. Although a previous scoping review did specifically investigate humanoid robots for older adult care, it was methodologically limited by its narrow search of only 2 databases and inclusion of literature only up to 2019 [19]. These limitations mean the review cannot account for the rapid technological advances and expanded evidence base in humanoid robotics over recent years. Consequently, the current evidence regarding humanoid robot applications for general populations of older adults remains fragmented and lacks an up-to-date, comprehensive synthesis to inform both research and practice.

Given this landscape, we used the scoping review framework of Arksey and O'Malley [20] to systematically map this emerging field. This approach optimally balances methodological rigor with the flexibility needed to (1) characterize current humanoid robot implementations and their care applications and (2) identify critical knowledge gaps requiring prioritized investigation. Our review thus provides both a comprehensive evidence synthesis and a roadmap for future research in this transformative domain.

## Methods

### Framework

This scoping review used a 5-stage methodological framework proposed by Arksey and O’Malley [20]. Prior to commencement, the study protocol was prospectively registered on the Open Science Framework on May 10, 2025, and is publicly accessible (registration DOI: 10.17605/OSF.IO/P2C8J) [21]. The review adhered strictly to this preregistered protocol without deviation. The conduct and reporting of this review strictly adhered to the PRISMA-ScR (Preferred Reporting Items for Systematic Reviews and Meta-Analyses Extension for Scoping Reviews) guidelines [22]. The completed PRISMA-ScR checklist is available in Checklist 1.

### Identifying the Research Question

This scoping review systematically examined the current evidence regarding the development and implementation of humanoid robots in health care for older adults. To comprehensively map the existing literature, we formulated the following broad yet focused research questions: (1) What types of humanoid robots have been implemented and what specific interventions have been delivered to older adult populations? (2) What is the reported feasibility, preliminary efficacy, and user experience (from older adults and caregivers/staff) associated with these interventions? (3) What critical gaps exist in the current literature that warrant focused attention in future research?

### Identifying Relevant Studies

The search strategy was designed to identify both published and unpublished sources, encompassing scientific and gray literature such as conference proceedings. This approach helped mitigate publication bias and capture emerging research, especially in the rapidly evolving field of robotics, where significant findings are often first presented outside traditional journals. An initial exploratory search was conducted in Web of Science to identify relevant articles on the topic. Key terms from the titles, abstracts, and index terms of these articles were extracted to inform the development of a comprehensive search strategy. The search combined robotics-related terms (eg, “humanoid robot,” “social robot,” “robot assist,” “human-robot interaction”) with terms referring to older adults (eg, “aged,” “older,” “elderly,” “geriatric”). A systematic 3-step search approach was used, covering literature published up to April 30, 2025. First, 7 major electronic databases including IEEE Xplore Digital Library, CINAHL, Cochrane Library, EMBASE, PubMed, Scopus, and Web of Science, were searched using predefined keywords and index terms (see Table S1 in Multimedia Appendix 1). Next, gray literature was sourced through the OpenGrey Repository. Finally, reference lists of key publications were examined, and citation chaining was performed to ensure thorough coverage of the relevant literature.

### Study Selection

**Figure 1.**
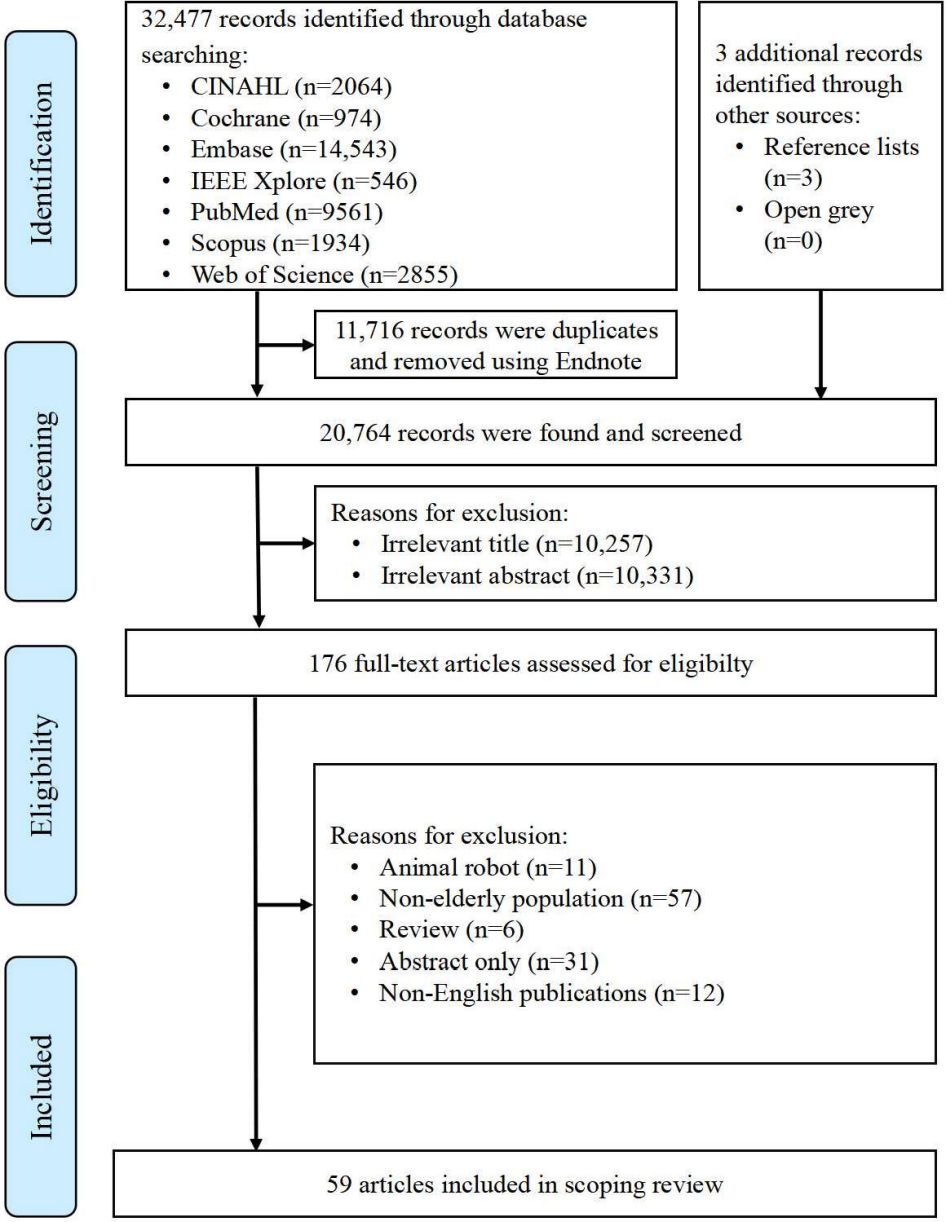
Flow diagram of the study retrieval and selection progress.

The review included original studies in which older adults (defined as individuals aged ≥60 years, aligning with the classification from the World Health Organization [WHO]) received any form of health care support through direct interaction with a physically embodied humanoid robot. We defined a humanoid robot as an autonomous or semi-autonomous machine with human-like morphology (eg, head, torso, arms; bipedal or wheeled mobility) and capable of performing social or physical tasks through human-like modalities such as speech, gesture, or facial expression. Studies that examined perspectives of other stakeholders (eg, care staff, family members, or health care professionals) within the same care context were also included, as these insights contribute to a holistic understanding of implementation and acceptability. For this review, health care is defined according to Merriam-Webster’s Dictionary as “efforts made to maintain, restore, or promote someone’s physical, mental, or emotional well-being especially when performed by trained and licensed professionals.” Our focus within this broad domain is on applications in both clinical settings (eg, hospitals) and long-term social care settings (eg, residential institutions and private homes). All study designs (eg, experimental, observational, qualitative) were considered, and both published and unpublished English-language literature was included without date restrictions. Studies were excluded if they (1) primarily focused on robotic hardware or software development without evaluating a health care application; (2) used nonembodied or simulated representations of robots (eg, video-based or computer-simulated interactions); (3) focused solely on fundamental human-robot interaction mechanisms (eg, laboratory studies of perception or dialogue systems without a health care context or outcome), as these did not address our objective of mapping evidence related to health care support for older adults; or (4 ) lacked available full text. The study followed a rigorous 4-phase selection process in accordance with PRISMA-ScR guidelines (Figure 1). After initial duplicate removal using EndNote 21 software, 2 independent reviewers conducted title and abstract screening followed by full-text evaluation. Inter-rater agreement was assessed using Cohen kappa, with a value >0.6 considered acceptable. The reviewers then reconciled their findings to ensure comprehensive coverage, with any discrepancies resolved through arbitration by a third reviewer.

### Charting the Data

We developed a standardized data extraction form aligned with the study objectives to systematically chart key information from included articles. Prior to full implementation, 2 independent reviewers piloted the data collection instrument using the first 5 eligible studies, refining the form through iterative discussion to enhance reliability. Extracted data elements encompassed (1) bibliographic details (authors, publication year, country of origin), (2) study characteristics (research setting, study design, study aims, sampling strategy, participant demographics), (3) outcome measures, and (4) significant findings. For humanoid robot–assisted health care, we specifically documented (1) core intervention components, (2) program content, and (3) implementation parameters (frequency and duration).

### Collating, Summarizing, and Reporting Results

Two independent reviewers conducted parallel analyses to ensure objectivity and minimize bias. Following thorough discussion and reconciliation of findings, the complete research team reviewed and validated the results. Using the PAGER (Patterns, Advances, Gaps, Evidence for practice, and Research recommendations) framework [23], we systematically identified 4 key patterns in the literature, along with their corresponding advances, gaps, practical implications, and future research directions. The PAGER framework is a structured methodological tool specifically designed for analyzing and reporting findings in scoping reviews, enhancing both the clarity and utility of the review output [23]. The acronym encompasses 5 interrelated analytical domains [23]: Patterns (key recurring themes emerging from the literature), Advances (notable developments within the recent evidence base), Gaps (critical limitations or underexplored areas), Evidence for practice (actionable insights for stakeholders), and Research recommendations (concrete suggestions for future research). A foundational step in applying the framework involves creating a patterning chart—a thematic table derived from inductive analysis of included studies—which visually organizes key findings and systematically guides the identification of advances, gaps, and resulting recommendations for research and practice [23].

## Results

### Study Selection and Characteristics

The study selection process, as depicted in the PRISMA-ScR flow diagram (Figure 1), commenced with the identification of 32,477 records from 8 electronic sources, with 3 additional records obtained from OpenGrey and manual reference searches. Following the removal of 11,716 duplicates using EndNote 21, we conducted initial screening of 20,764 records. Title and abstract review led to the exclusion of 20,588 records that did not satisfy the inclusion criteria. The analysis yielded a Cohen kappa value of 0.88 for inter-rater agreement. Subsequently, 176 full-text articles underwent comprehensive eligibility assessment, culminating in the inclusion of 59 studies for final analysis (see Table S2 in Multimedia Appendix 1).

Table 1 and Tables S3 and S4 in Multimedia Appendix 1 present the key characteristics of the included studies. Publication years spanned from 2012 onward, showing distinct temporal clustering with 10 studies (10/59, 17%) published before 2015, 18 studies (18/59, 31%) published between 2016 and 2020, and 31 studies (31/59, 53%) published after 2020. The publications originated from research teams across multiple regions: Europe (n=20), Asia (n=15), North America (n=7), and Oceania (n=4), with an additional 13 studies representing multinational collaborations. The majority (49/59, 83%) were conducted in real-world settings—defined for this review as environments where care is routinely provided with minimal researcher intervention, as opposed to controlled laboratory or research center environments—while laboratory environments accounted for 10% (6/59), and 5% (3/59) did not specify their location. Across the included studies, participants primarily comprised older adults (59/59, 100%), with a subset also involving informal caregivers (5/59, 9%), formal caregivers (14/59, 24%) and, in 3 studies, individuals categorized as “others.” Methodologically, 34 studies (34/59, 58%) had small sample sizes (n≤25). Among the 59 studies, 26 (44%) adopted a quantitative design, 22 (37%) used a mixed methods approach, and 11 (19%) used qualitative methodologies. Within the quantitative subset, designs varied considerably, encompassing 6 observational (cross-sectional) studies, 4 randomized controlled trials, 5 single-case/within-subjects experimental designs, 7 quasi-experimental (nonrandomized, group-based) studies, and 4 pre-post studies. Participant characteristics revealed female predominance (>50%) in 32 studies (32/59, 54%), while 16 studies (16/59, 27%) omitted gender data. Notably, 27 studies (27/59, 46%) incorporated participants with cognitive impairment.

**Table 1.** Characteristics of included studies (n=59).

Characteristics			Studies, n (%)	Study references
Publication year				
2021‐2025			31 (53)	[8-12,24-49]
2016‐2020			18 (31)	[50-67]
2012‐2015			10 (17)	[68-77]
Country				
Europe				
Italy			6 (10)	[8,24,30,33,34,48]
France			5 (9)	[27,40,43,44,73]
Germany			2 (3)	[10,69]
Poland			5 (9)	[32,35,36,45,60]
Finland			1 (2)	[51]
Spain			1 (2)	[68]
Asia				
Japan			8 (14)	[28,42,56,57,63,64,67,77]
China			4 (7)	[37,52-54]
South Korea			2 (3)	[11,47]
Israel			1 (2)	[62]
North America				
United States			5 (9)	[12,39,58,65,75]
Canada			2 (3)	[9,72]
Oceania				
New Zealand			1 (2)	[70]
Australia			3 (5)	[66,74,76]
Multinational collaboration			13 (22)	[25,26,29,31,38,40,46,49,50,55,59,61,71]
Study design				
Quantitative study			26 (44)	[9,11,12,27,28,30,32,33,35,36,41,42,45,47,48,52-54,57,59,60,63,64,68,72,75,77]
Observational designs				
Cross-sectional study			6 (23)	[32,35,36,45,63,72]
Experimental designs				
Randomized controlled trial			4 (15)	[47,52,54,77]
Single-case experimental design/within-subjects design			5 (19)	[41,42,57,64,75]
Quasi-experimental studies				
Quasi-experimental study (group, nonrandomized)			7 (27)	[9,11,27,30,48,60,68]
Pre-post study			4 (15)	[12,28,33,59]
Qualitative study			11 (19)	[8,25,29,31,37,49,51,53,55,56,62]
Mixed method design			22 (37)	[10,24,26,34,38-40,43,44,46,50,58,61,65-67,69-71,73,74,76]
Setting				
Day care center			1 (2)	[8]
Clinic			3 (5)	[24,34,48]
Home			6 (10)	[25,31,37,69,70,77]
Community			5 (9)	[29,38,39,41,47]
Hospital			7 (12)	[33,43,44,50,53,60,75]
Long-term care facility			15 (25)	[9,12,26-28,40,46,54,56-58,63,65,66,76]
University			1 (2)	[52]
Multiple sites			12 (20)	[32,35,36,42,45,49,51,55,59,64,68,74]
Laboratory			6 (10)	[10,11,61,62,71,73]
Not specified			3 (5)	[30,67,72]
Key robot functions in health care				
Social interaction			23 (39)	[12,26,28,32,34,39,45,51-53,55,58,59,61,63,65,66,70-74,76]
Cognitive training			15 (25)	[8,24,34,36,37,41,46-48,55,59,62,68,72,73]
Physical activity facilitation			11 (19)	[9,25,26,32,33,38,49,52,56,57,64]
Health care support			10 (17)	[29,32,37,38,60,65,70,74-76]
Daily life support			8 (14)	[37,38,43,45,60,61,66,69]
Companionship			8 (14)	[29,37-39,54,65,69,71]
Health monitoring			4 (7)	[32,59,69,71]
Communication			4 (7)	[10,49,63,77]
Memory training			3 (5)	[27,30,50]
Health education			3 (5)	[11,44,67]
Entertainment			3 (5)	[43,66,70]
Health coaching			2 (3)	[29,31]
Rehabilitation			2 (3)	[51,75]
Leisure facilitation			1 (2)	[42]
Handover			1 (2)	[40]
Emergency assistance			1 (2)	[71]

### Description of Humanoid Robot–Assisted Health Care for Older Adults

See Table S5 in Multimedia Appendix 1 for details on humanoid robot–assisted health care interventions. Across the included studies, 25 distinct humanoid robots were used, with Pepper being the most frequently used (n=15), followed by Nao (n=9), TIAGo (n=4), Kabochan (n=3), and Matilda (n=3). The remaining robots were each used in 1 or 2 studies. As detailed in Table 1, the interventions covered 16 key functional domains, with the number of studies per domain ranging from 1 to 23. The most prevalent functions were social interaction (23/59, 39%), cognitive training (15/59, 25%), and physical activity facilitation (11/59, 19%). Among the 59 studies analyzed, 4 did not report intervention duration. Of the remaining 55 studies (55/59, 93%), the majority (30/55, 55%) lasted 1 week or less; 11% (6/55) lasted between 1 week and 4 weeks; and 35% (19/55) extended beyond 4 weeks, including 4 studies (4/55, 7%) that continued for more than 24 weeks.

### Patterns of Research Findings

We identified 4 key PAGER patterns of research findings (see Table 2; also see Multimedia Appendix 2 for a detailed analysis of all included studies).

**Table 2.** PAGER (Patterns, Advances, Gaps, Evidence for practice, and Research recommendations) framework.

Pattern	Advances	Gaps	Evidence for practice	Research recommendations
Effects, perceptions, and experience of humanoid robots	Preliminary studies indicate the potential of humanoid robots to be effective in care for older adults, with many reporting positive user acceptance and experiences.	Lack of randomized controlled trials, short-duration humanoid robot applications, and small sample sizes and imbalanced population characteristics	Emerging studies indicate that humanoid robots may have the potential to improve cognitive, physical, and psychosocial health in aging populations.	Prioritize conducting rigorously designed randomized controlled trials with larger, more balanced samples to evaluate the clinical effectiveness of longer-term humanoid robot interventions while simultaneously assessing older users’ perceptions and experiences.
Preference, expectations, and facilitators for humanoid robots	Heterogeneous and individualized preferences have been observed among older adults regarding humanoid robots’ appearance, functionality, and interaction modalities.	Small sample sizes and imbalanced population characteristics	Available findings suggest that involving older adults in the design process to tailor a robot’s appearance and functions to individual preferences could enhance its acceptance and utility in care settings.	Use representative sampling strategies and involve cross-disciplinary teams to develop and evaluate personalized robotic interventions, with specific attention to distinguishing between initial novelty effects and sustained engagement over time.
Implementation barriers and challenges	The adoption of humanoid robots in care for older adults may face multifaceted barriers across technological, user-specific, and contextual dimensions.	Lack of multistakeholder perspectives	To facilitate implementation, practice should focus on developing robots with senior-friendly interfaces and complementing their introduction with tailored digital literacy programs for older users and their caregivers.	Adopt a multistakeholder analytical framework, gathering and integrating perspectives from older adults, formal and informal caregivers, health care professionals, policymakers, and technology developers to identify comprehensive implementation strategies.
Determinants of user experiences and outcomes	Available evidence suggests that user experiences and outcomes with humanoid robots may be influenced by user-related factors (eg, demographics, health status, and psychological state) and robot-related characteristics (eg, appearance, personality, and interaction methods).	Small sample sizes and imbalanced population characteristics as well as narrow factor consideration (eg, omitting environmental and contextual variables)	Emerging studies point to the importance of considering user experiences and outcomes as key factors in shaping effective human-robot interactions.	Expand sample sizes and systematically examine a broader range of potential determinants, including environmental (eg, home vs institutional setting) and contextual factors, to better understand what shapes user experience and outcomes.

### Effects, Perceptions, and Experience With Humanoid Robots

Research indicates that humanoid robot interventions can yield effects across psychosocial, cognitive, functional, and behavioral domains. A frequently reported psychosocial benefit is enhanced social interaction [8,11,12,25,26,29,37,38,42,43,46,48,51-53,55,58-60,63,71], with associated reductions in loneliness [12,29,37,45]. Improvements in mood and emotional states [8,12,24,25,28,39,42,46,55,59,65,77], alongside decreases in stress, depression, and anxiety [8,12,24,30,39,46,47,58,59,65], have also been observed, although not all studies report significant changes in these areas [58]. In cognitive and functional domains, evidence suggests potential for cognitive stimulation [8,24,29,30,41,46,47,50,55,68,74-77], enhanced task performance [24,41,48,64], and improved limb function [33,56,75]. Regarding health behaviors, some interventions show promise for promoting medication adherence [29,67,70], physical activity [29,31,33,51,56,76], better dietary habits [76], and sleep quality [77]. It is noteworthy that, in specific task-oriented contexts, robotic performance has been perceived as inferior to human care [27]. The sustainability of these positive effects is a critical concern, with evidence pointing to potential attenuation over time [38] or a lack of significant long-term improvement in outcomes such as core dementia symptoms, depression, and quality of life [38,54,58,59].

Perceptions of humanoid robots among older adults and formal oor informal caregivers are heterogeneous, encompassing both positive dimensions and reservations. A substantial body of literature reports favorable perceptions from older adults across multiple dimensions, including acceptability [8,25,26,29,30,38,44-46,49,52,53,60,61,64-66,69-74,76], usability [24,30,38,43,44,50,52,60,69,71], trust [31,64,71,73], satisfaction [11,33,40,43,61], and active or positive attitudes [8,10,24,35,37,43,46,51,55,57,61,65,66,69,72,75,76]. In practice, this manifests as increased user engagement and motivation [8,12,24-26,33,37,39,44,46,48,50,55,58,66,74,76,77] and strengthened perceived social support [59] in some contexts. However, user acceptance is not universal. Some older adults perceive robots as “too technical” or “lacking a human touch” [51], a challenge exacerbated for those with sensory impairments [51]. Caregiver perceptions are similarly mixed. Although some studies indicated a potential reduction in caregiver workload [51,54,60,76], others report an increased burden due to the need to assist with the technology [46]. Formal caregivers frequently cite practical barriers, including the additional time required for operation, difficulties in workflow integration, and, in some cases, negative attitudes toward the technology’s value or appropriateness [51]. Cost-effectiveness is another point of skepticism, raised both by older adults [25] and implicitly within the context of practical implementation challenges.

The lived experience of interacting with these robots underscores several critical considerations. Safety and adverse events, though infrequent, warrant attention. These range from user-expressed safety concerns [57] to documented incidents such as medication errors [38] and adverse psychological reactions leading to study withdrawal [54]. Ethical concerns prominently feature issues of autonomy and privacy, with a noted divergence in emphasis between older adults (prioritizing user control) and caregivers (exercising more caution) [35]. Furthermore, equity and access emerge as significant issues, exemplified by cases where robot design (eg, language support) inadvertently created barriers for participants, highlighting risks of exacerbating the digital divide [46].

### Preferences, Expectations, and Facilitators for Humanoid Robots

This synthesis reveals a clear pattern in older adults’ preferences for humanoid robots. Older adults show marked preference for humanoid robots’ health management functions, especially medication reminders and daily routine notifications [25,43]. Assistive functions like safety monitoring and health reminders consistently receive higher utility ratings than purely social features [45]. At the same time, entertainment-oriented functions such as music playback and memory recall features exhibit high engagement frequency, with studies showing these applications significantly improve mood states and facilitate positive reminiscence [55]. These core functionalities are typically complemented by basic conversational interactions including weather inquiries and casual chats in real-world usage scenarios [37,65].

Beyond functionality, user expectations shape perception and interaction. Users perceive the empathetic version of Ryan robot as more attractive, friendly, and compassionate than its nonempathetic counterpart [39]. Notably, multisensory integration—combining visual, auditory, and tactile stimuli—elicited significantly stronger happiness responses than unimodal (visual-only) or bimodal (visual-auditory) stimulation approaches [28]. Some evidence suggests that older adults may prefer in-person human-robot interaction over remote formats [9]. In studies of walking assistance, participants have been observed to position the robot more as a “navigational guide” rather than a “walking companion” [64]. Regarding humanoid robotic appearance expectations, research indicates older adults expect larger-sized robots [25] but typically reject those taller than themselves [43]. Their appearance expectations encompass a “smiling” and “handsome” design with fluid movements [43], along with friendly aesthetics, strong interactivity, simple operation modes, and integrated verbal-physical interactions [44].

Research documents older adults’ functional requests for humanoid robotic systems, which serve as key facilitators for adoption. These emphasize improved interaction quality (enhanced speech clarity, real-time responses, and personality adaptation) [25,31,37], personalized health features (tailored exercise programs and accurate reminders) [38], expanded utility (medication management and emergency services) [37,43], and technical enhancements (advanced voice recognition and adaptive mobility) [55,57]. Although users show interest in customizable cognitive activities and varied applications [65,72], they consistently express reservations about privacy-sensitive functions such as bathing assistance [61].

### Implementation Barriers and Challenges

From the machine or technical perspective, limitations manifest across several operational domains. Hardware constraints significantly impair functional capacity, notably in fine motor manipulation [40] and ambulatory assistance [56]. System reliability issues are characterized by intermittent connectivity [43] and unanticipated software termination [26], while interaction protocols demonstrate inadequate natural language processing capabilities [27,49,62]. Sensory apparatuses exhibit suboptimal performance in acoustically challenging environments [55] and human motion detection [43]. User interface implementations frequently present usability barriers, including unintuitive volume modulation controls and instances of erroneous information dissemination (eg, inaccurate meteorological alerts) or unintended nocturnal activation [38]. Crucially, current systems predominantly operate under supervised autonomy paradigms [12], where robots execute predetermined tasks but remain reliant on human oversight and intervention for initiation, safety, and context management rather than achieving genuine operational independence [49].

From the human or user perspective, adoption barriers emerged across 3 principal dimensions. Physical limitations include difficulties engaging with activation protocols [31] and potential safety hazards from unpredicted robotic motions [49]. Psychological factors encompass both initial technophobia [46] and entrenched self-perceptions of digital incompetence [73], necessitating extended skill acquisition periods [47]. Sociocultural concerns involve professional displacement anxieties among care staff [51], diminished interpersonal engagement [73], and surveillance-related ethical dilemmas [49]. Most significantly, prolonged use of these systems may lead to a decline in users’ own abilities due to lack of practice and could also undermine the human essence of caregiving [73].

Implementation challenges intersect both perspectives, as even technically proficient systems demand substantial care protocol modifications and extensive personnel training [51], with economic viability assessments challenging the utility of specific design implementations [25].

### Determinants of User Experiences and Outcomes

Older adults’ experiences and outcomes with humanoid robots are influenced by multiple factors, with significant individual variations reported across studies. Available findings on demographic characteristics are mixed. For instance, one study suggested that men might be more inclined than women to perceive robots as companions [35], while another found that women exhibited greater cognitive improvement in a specific robot-assisted cognitive training program [47]; however, other research observed no significant gender differences in well-being outcomes [42]. Similarly, although age was not found to significantly affect older adults’ attitudes toward robots or their well-being in one study [42], another reported more pronounced cognitive benefits from training among participants aged 75 years and older [47]. Regarding education level, findings present a dual pattern. One study reported that individuals with higher education levels exhibit lower acceptance of robots’ social functions [36], whereas a different study found that those with lower education levels showed more significant cognitive improvement in a training context [47]. In terms of health status, some evidence indicates that older adults with less independence in daily living may display greater reliance on robotic assistance [35], and those with higher dependency levels have been associated with moderately reduced well-being scores in one study [42]. Psychological states appear to influence interaction tendencies. For example, one study observed that individuals exhibiting agitation or psychotic symptoms showed stronger engagement willingness than those with depressive or apathetic symptoms [54]; the same study also noted that hearing impairment and sleep deprivation were linked to significantly impaired interaction outcomes [54]. At the interaction design level, findings from a specific study indicated that multimodal (voice-gesture) interaction was most effective at enhancing emotional connection and familiarity [11]. In another study, introverted robot personalities were perceived as more natural in interaction, whereas extroverted robots were considered more entertaining but slightly intrusive [34]. Furthermore, key robot perception dimensions—such as anthropomorphism, animacy, likability, and perceived intelligence—have been found to show significant correlations with functional performance in one study [36].

## Discussion

This scoping review provides a systematic examination of humanoid robot–assisted health care support for older adults, analyzing 59 studies that document 25 distinct humanoid robot models currently implemented in both real-world and laboratory settings. Using the PAGER framework as our analytical lens, we identified and organized the extant literature into 4 conceptually distinct patterns. These emergent patterns are detailed in the following sections.

### Effects, Perceptions, and Experience With Humanoid Robots

This scoping review aligns with previous findings [19] by demonstrating the multifaceted applications of humanoid robots across various domains of older adult care, encompassing social interaction, cognitive stimulation, physical activity facilitation, and activities of daily living support. Evidence indicates these robotic solutions can successfully promote social interaction, deliver cognitive enrichment, and enhance motor function in older populations. Notably, older adults have demonstrated favorable reception of humanoid robots, expressing positive evaluations of their usability and satisfaction levels while exhibiting constructive attitudes toward this technology. Together, these findings underscore the considerable promise of humanoid robots for innovative implementations in older adult care. Nevertheless, the methodological heterogeneity and specific limitations of the included studies restrict the strength and generalizability of our inferences. In line with existing literature [14], a recurrent methodological limitation across the reviewed literature is the prevalent use of studies with small sample sizes. This issue is frequently compounded by significant gender imbalances, typically skewed toward female participants, and a predominant research focus on older adults with cognitive impairments, potentially limiting the generalizability of findings to the wider, more heterogeneous aging population. Methodologically, the field shows a striking lack of randomized controlled trials—the gold standard for clinical evidence—compounded by typically short intervention durations that fail to capture long-term effects. Additionally, approximately 10% of the included studies were conducted in laboratory settings, which may further restrict the ecological validity and real-world applicability of the reported outcomes. In terms of functionality, current humanoid robot applications remain narrowly focused on cognitive training and social interaction, as confirmed by both our findings and prior research [78]. This limited scope stands in stark contrast to the broader spectrum of care needs documented in the literature [79], which includes essential daily living supports like fall prevention, feeding assistance, toileting, bathing, and position changing. To advance the field, future work should prioritize the development of more versatile robotic systems while conducting rigorous, large-scale multicenter trials with extended observation periods to fully assess the potential of humanoid robots in older adult care settings.

### Preference, Expectations, and Facilitators for Humanoid Robots

This scoping review elucidates older adults’ preferences and expectations concerning humanoid robots, encompassing functionality, design features, and physical appearance, while also capturing their developmental aspirations for this technology. Our analysis reveals that seniors exhibit complex, multidimensional expectations that are not only highly personalized but also consistently oriented toward robotic systems with expanded capabilities, enhanced stability, sophisticated intelligence, and greater autonomy. These combined demands, which encompass both personalization and high-performance functionality, directly translate into a core technical requirement: Assistive robots must be highly adaptable to diverse home environments and individualized user needs. Achieving this necessary level of adaptability in a cost-effective manner represents a significant technical hurdle requiring advances in sensors, machine learning algorithms, and robotic hardware [80]. However, the current evidence base presents notable methodological constraints. Many included studies have similar methodological limitations as noted in the previous section, particularly regarding sample representativeness. Building upon nursing science’s established tradition of person-centered care [81,82], we propose an integrated development framework that synergizes technological innovation with individualized implementation strategies. This approach necessitates multidisciplinary collaboration among gerontologists, engineers, designers, and health care professionals to advance humanoid robot capabilities, particularly in modular customization, natural interaction algorithms, and reliable multicontext functionality [83]. Parallel to these technical advancements, comprehensive predeployment assessments incorporating detailed geriatric evaluations and user profiling will be essential to ensure optimal alignment between robotic solutions and individual user requirements, thereby maximizing both clinical efficacy and resource efficiency.

### Implementation Barriers and Challenges

Similar to prior studies [84,85], we demonstrate that widespread adoption of humanoid robots faces substantial barriers from technical, human, systemic, and sociocultural perspectives. A critical gap in the included literature is its overemphasis on end user experiences while neglecting other essential stakeholders including family caregivers, health care providers, policymakers, and technology developers [18]. This narrow perspective creates an incomplete framework for implementation, which must account for seniors’ needs alongside caregiver workflows, institutional policies, and financial sustainability. Furthermore, the implementation context is profoundly shaped by culture-related factors that are often underexamined. These include deeply held social norms regarding the appropriateness of delegating intimate care tasks to machines, varying ethical frameworks across societies for balancing technological efficiency with the preservation of human dignity in care, and differing levels of societal trust in automation. To address these limitations, future research should use a comprehensive approach that engages all relevant stakeholders and explicitly investigates the cultural contexts of deployment settings. For older adult users specifically, implementation strategies should use a phased approach that includes assessing initial perceptions, clarifying concepts, addressing concerns, and demonstrating benefits to enhance acceptance [86]. Structured communication channels and collaborative guideline development can help mitigate risks while fostering stakeholder engagement. From a technical perspective, developing adaptable systems for diverse environments and user needs remains paramount, though cost-effective solutions will require advances in sensors, machine learning, and hardware design. Ethical considerations must go beyond minimum standards, implementing robust data consent protocols that exceed basic International Organization for Standardization requirements [87,88]. Successful implementation further requires addressing affordability to ensure equitable access, developing comprehensive training programs to optimize use, and planning workforce transitions that include retraining opportunities. Culturally appropriate design remains essential for widespread adoption [87]. A successful integration strategy must therefore balance technological innovation with human-centered design and organizational preparedness.

### Determinants of User Experiences and Outcomes

Our scoping review reveals that the effectiveness of humanoid robots is determined by both user-related factors (including demographics, health status, and psychological characteristics) and robot-related features (such as appearance, personality traits, and interaction modalities). The studies included in this review exhibit notable methodological constraints, particularly regarding the common issue of limited sample sizes with demographic imbalances and insufficient examination of contextual and environmental variables. Subsequent investigations should prioritize more representative participant cohorts and systematically evaluate the complete spectrum of influential factors to elucidate the key determinants of robot-assisted older adult care. Critically, as emphasized throughout this review, transitioning from standardized implementations to tailored, individualized approaches represents an essential paradigm shift for optimizing humanoid robot integration in older adult health care settings.

### Strengths and Limitations

To our knowledge, this represents the most comprehensive and methodologically rigorous scoping review to date examining the applications of humanoid robots in older adult health care. Our study, which was prospectively registered, used the established 5-stage methodological framework by Arksey and O’Malley [20] and adhered to PRISMA-ScR guidelines for transparent reporting. Notwithstanding these strengths, several limitations warrant consideration. First, although our systematic 3-step search strategy identified 59 relevant articles across 7 databases and gray literature sources, we acknowledge the possibility of having missed pertinent studies, particularly those where humanoid robotics applications were not explicitly stated in titles or abstracts. The exclusion of internet searches may have further contributed to this limitation. Second, our English language restriction may have introduced selection bias by excluding potentially relevant non-English publications. Finally, the scope of this review did not encompass certain specialized domains such as robotic surgery or monitoring systems using humanoid platforms.

### Implications

This scoping review underscores the considerable promise of humanoid robots in enhancing health care delivery for aging populations, positioning them as a viable solution to mitigate future older adult care challenges. The field has evolved from early locomotion-focused platforms to today’s interactive, AI-enhanced systems capable of complex social and assistive functions. Future advancements are expected to encompass greater personalization, multimodal sensing, expanded physical assistance capabilities, and stronger ethical frameworks to support real-world integration [7]. However, the seamless integration of this technology into the daily lives of older adults remains a complex challenge that requires careful consideration. The findings reveal that the needs and expectations of older adults are multifaceted, demanding highly personalized approaches. Successful implementation of humanoid robots in older adult care must extend beyond technological functionality to incorporate the perspectives of all stakeholders—older adults themselves, family members, professional caregivers, health care staff, institutional leaders, and policymakers. A one-size-fits-all model would be insufficient; instead, adaptable solutions tailored to individual needs are essential to enhance care quality and optimize resource efficiency. However, achieving this personalization at scale necessitates addressing its economic viability. Research and policy must concurrently focus on reducing costs through modular design, scalable software personalization, and innovative funding or reimbursement models to prevent the benefits of personalized robotic care from becoming a privilege of the few. Moreover, the effectiveness of humanoid robots in older adult care is shaped by a dynamic interplay of technological, human, environmental, and ethical factors. Advancing this field requires interdisciplinary collaboration across health sciences, computer science, and engineering, alongside the establishment of standardized frameworks to improve robotic functionality, safety, and autonomy while minimizing risks. Equally critical is the development of older adult–friendly interfaces and targeted training programs to improve digital literacy among older adults. Professional caregivers also need structured instruction to facilitate effective human-robot collaboration in care settings. Simultaneously, ethical concerns—including privacy protection, autonomy preservation, and the establishment of appropriate human-robot boundaries—must be systematically addressed to ensure responsible adoption and sustained trust in robotic care solutions. Although the promise of humanoid robots in older adult care is evident, their long-term viability hinges on addressing these multifaceted challenges through coordinated efforts across technological innovation, user-centered design, workforce training, and ethical governance. Finally, future rigorous randomized controlled trials should prioritize the inclusion of well-defined control groups to precisely isolate the unique effect of humanoid robot interventions. Appropriate control conditions may include, but are not limited to (1) standard care or treatment as usual to establish baseline effectiveness, (2) interventions delivered by human caregivers or companions to benchmark robotic performance against the human touch, and (3) other technological or robotic interfaces (eg, tablet apps, nonhumanoid robots) to distinguish the impact of humanoid form factors and interaction modalities from general technology effects. Such comparisons are essential for determining not just if humanoid robots are effective but also for whom, in what contexts, and relative to what alternatives.

### Conclusions

This scoping review systematically mapped the literature on the implementation of humanoid robots in health care settings for older adults, identifying 25 distinct models currently in use. By applying the PAGER framework, the review delineated 4 key thematic patterns within the evidence: (1) effects, perceptions, and experiences; (2) preferences, expectations, and facilitators; (3) implementation barriers and challenges; and (4) determinants of user experiences and outcomes. The available evidence suggests that deployments to date have focused largely on social interaction and cognitive stimulation, with many studies reporting favorable initial user acceptance and experience. Findings also point to individualized user preferences and highlight that adoption is likely influenced by a complex interplay of technical, user-specific, and contextual factors. The analysis underscores persistent evidence gaps—most notably a lack of robust experimental designs, long-term outcome data, and studies conducted in ecologically valid settings with representative populations. Future research should, therefore, prioritize large-scale, real-world trials with diverse older adult populations to evaluate the long-term efficacy and implementation of multifunctional humanoid robots across the full spectrum of care needs. Ultimately, addressing these research gaps will be crucial for overcoming the technical, user-related, and systemic challenges to successful integration.

## Supplementary material

10.2196/83849 Multimedia Appendix 1 Index, keywords, and list and characteristics of included studies.

10.2196/83849 Multimedia Appendix 2 Detailed PAGER (Patterns, Advances, Gaps, Evidence for practice, and Research recommendations) framework analysis of included studies.

10.2196/83849 Checklist 1 PRISMA-ScR (Preferred Reporting Items for Systematic Reviews and Meta-Analyses Extension for Scoping Reviews) checklist.

## Abbreviations

PAGER: Patterns, Advances, Gaps, Evidence for practice, and Research recommendations
PRISMA-ScR: Preferred Reporting Items for Systematic Reviews and Meta-Analyses Extension for Scoping Reviews
WHO: World Health Organization
